# Spiked GBS: a unified, open platform for single marker genotyping and whole-genome profiling

**DOI:** 10.1186/s12864-015-1404-9

**Published:** 2015-03-28

**Authors:** Trevor W Rife, Shuangye Wu, Robert L Bowden, Jesse A Poland

**Affiliations:** Interdepartmental Genetics, Kansas State University, 4024 Throckmorton Hall, Manhattan, KS 66506 USA; Wheat Genetics Resource Center, Department of Plant Pathology, Kansas State University, 4024 Throckmorton Hall, Manhattan, KS 66506 USA; USDA-ARS, Hard Winter Wheat Genetics Research Unit, 4008 Throckmorton Hall, Manhattan, KS 66506 USA; Department of Plant Pathology, Kansas State University, Manhattan, KS 66506 USA

**Keywords:** Plant breeding, Wheat, Marker platform, Genotyping-by-sequencing

## Abstract

**Background:**

In plant breeding, there are two primary applications for DNA markers in selection: 1) selection of known genes using a single marker assay (marker-assisted selection; MAS); and 2) whole-genome profiling and prediction (genomic selection; GS). Typically, marker platforms have addressed only one of these objectives.

**Results:**

We have developed spiked genotyping-by-sequencing (sGBS), which combines targeted amplicon sequencing with reduced representation genotyping-by-sequencing. To minimize the cost of targeted assays, we utilize a small percent of sequencing capacity available in runs of GBS libraries to “spike” amplified targets of *a priori* alleles tagged with a different set of unique barcodes. This open platform allows multiple, single-target loci to be assayed while simultaneously generating a whole-genome profile. This dual-genotyping approach allows different sets of samples to be evaluated for single markers or whole genome-profiling. Here, we report the application of sGBS on a winter wheat panel that was screened for converted KASP markers and newly-designed markers targeting known polymorphisms in the leaf rust resistance gene *Lr34*.

**Conclusions:**

The flexibility and low-cost of sGBS will enable a range of applications across genetics research. Specifically in breeding applications, the sGBS approach will allow breeders to obtain a whole-genome profile of important individuals while simultaneously targeting specific genes for a range of selection strategies across the breeding program.

**Electronic supplementary material:**

The online version of this article (doi:10.1186/s12864-015-1404-9) contains supplementary material, which is available to authorized users.

## Background

Progress in plant breeding focuses on the rapid development of new cultivars with improved attributes. Molecular markers allow breeders to characterize specific lines without the need for laborious and time-consuming phenotyping. Marker-assisted selection (MAS) is used in plant breeding to identify the allele present at a specific locus, allowing the breeder to select based on genotype [1]. MAS has been used for plant breeding in many crops to identify specific individuals with known genes of interest [2-4], primarily to target large-effect, single targets [5,6]. Since each locus is generally genotyped independently, breeders tend to consider *per data point* costs when utilizing MAS within breeding programs.

Contemporary marker technologies for assaying single targets that are often used with MAS include KASP, targeted amplicon sequencing, and SNP arrays. KASP (Kompetitive Allele Specific PCR) is a uniplex, fluorescence-based single nucleotide genotyping technology that utilizes allele-specific oligo extension [7]. KASP markers have been used for breeding, QTL mapping, and are the main genotyping platform for the Generation Challenge Program at CIMMYT [7]. The arrival of inexpensive sequencing has led to the development of economical sequence-based genotyping approaches. Targeted amplicon sequencing (TAS) amplifies known gene targets and attaches a barcode in a second PCR reaction for multiplexing [8]. Samples are pooled, sequenced, and analyzed by parsing the sample-specific barcode and then identifying *a priori* or newly discovered variants [8,9]. Using a targeted amplicon approach, Bybee et al. [8] specifically looked at genes useful for phylogenetic analysis. TAS was further extended to a single PCR reaction that utilized linker sequences which allowed common target primers and a single set of barcoded primers to be utilized across distinct samples and loci [10].

Complementary to assaying single loci for MAS, whole-genome profiling can be utilized for genomic section, QTL mapping, and diversity analysis [11]. Whole-genome profiling approaches focus on assaying large numbers of markers while reducing the *per sample* cost [12]. Two common whole-genome profiling methods are SNP arrays and genotyping-by-sequencing (GBS). SNP arrays are comprised of a large number of known polymorphisms that allow an individual to be genotyped at all sites simultaneously which reduces the overall cost per data point [13]. SNP arrays have been used across a range of species to characterize diversity [14,15] and for association mapping [16]. SNP arrays tend to be robust marker platforms but can have limitations, including the inability to target loci that were not included during the array development (i.e. ascertainment bias) and a relatively high per-sample cost.

GBS is a reduced representation whole-genome profiling strategy that leverages rapidly dropping sequencing cost and increasing output. Multiplexing samples with DNA barcodes greatly reduces the per sample cost [17,18]. GBS is one of several reduced representation marker platforms to take advantage of second-generation sequencing platforms that produce enormous amounts of sequence [12,19]. However, since many samples are sequenced together to minimize cost, the reduced sequencing coverage per sample often results in higher levels of missing data. Since sequencing is only targeted to regions flanking restriction sites, GBS is unable to directly ascertain specific loci, leading to considerable informatics challenges when used in MAS.

Spiked genotyping-by-sequencing (sGBS) takes advantage of the abundant sequencing output by combining reduced representation GBS libraries with multiple, targeted amplicons. sGBS assesses known alleles via targeted amplicon sequencing and individual genotypes are determined by allele frequency counts. Multiple loci can be assayed concurrently since genotyping relies on the direct sequence output. A similar approach to sGBS was developed by Wells et al. [20] that utilizes sequencing-based variant detection by barcoding amplicons. sGBS is more economical since it uses only a small fraction of available sequencing capacity, the majority of which is simultaneously being used to generate independent, whole-genome profiles. By combining both approaches, breeders and geneticists are able to employ multi-faceted selection strategies and marker assays with nominal resource expenditure.

To evaluate this approach, we performed sGBS on a winter wheat panel that was screened for six converted KASP markers, four known polymorphisms in the leaf rust resistance gene *Lr34*, and one newly-designed marker targeting a known deletion in *Lr34*.

## Methods

### Plant material

A panel of 153 diverse, advanced wheat lines (Additional file 1: Table S1) was assembled and DNA was extracted from seedling leaf tissue using a BioSprint 96 DNA Plant Kit (Qiagen). DNA was quantified in plates using PicoGreen and concentrations were normalized to 20 ng/μL.

### Markers

Eleven single nucleotide markers were tested for the sGBS approach. Six of the markers were converted from a set of the KASP core markers: BS00023148, BS00083385, BS00150192, BS00067189, BS00088726, and BS00089969 [21]. Four of the markers were developed from previously designed *Lr34* KASP markers: Lr34exon11kasp, Lr34exon12kasp, Lr34intron4kasp, and Lr34exon22kasp [22]. The ‘Lr34exon11’ marker from Lagudah et al. [22] was also adapted for sGBS, by targeting a 3 bp insertion in exon 11, indicative of a non-functional allele (Lr34 minus). All primer and allele sequences are provided in Additional file 2: Table S2. Two of the markers from the KASP core collection did not amplify (BS00067189 and BS00088726) and were not included in the subsequent analysis.

### Primer design

**Figure 1:**
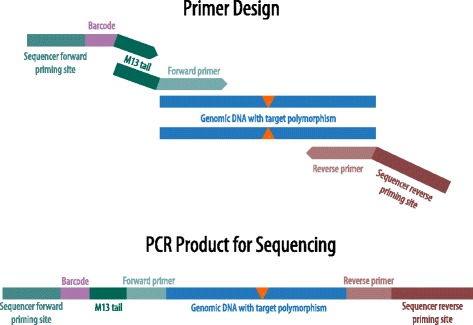
**Primer and amplicon construction.** The first round of PCR uses a forward primer containing the M13 sequence to amplify the target region. The second round of PCR extends from the M13 tail and incorporates a unique barcode, leading to a final product containing the sequencer primers, barcode, M13 sequence, and polymorphic target.

Primers were designed to amplify the full sequencing construct in a single PCR reaction (Figure 1). A set of 384 unique barcoded primers was developed for multiplexing and to differentiate spiked amplicons from GBS reads (Additional file 3: Table S3). Each barcode primer contains a sequencing platform forward priming site, a unique 10-base barcode, and a M13 tail sequence (Figure 1). These were combined with allele-specific primers that also included the M13 tail sequence on the forward primer [23]. The allele-specific reverse primer includes both the flanking sequence reverse primer and the sequencer-specific reverse priming site. Incorporating the M13 tail design on both the barcoded primer and allele-specific primer enables the utilization of the same set of barcode oligos for any target sequence, amortizing the cost of oligo synthesis for barcodes across many samples. The alternative of making barcoded allele specific primers for each target locus would be cost-prohibitive.

KASP markers were converted to primers for sGBS by removing the selective base on the end of each forward primer, effectively creating a single, common forward primer for each locus rather than the two allele-specific primers used for KASP genotyping. Integrating the respective M13 and reverse Ion Torrent sequences on the primer pair made the KASP primer sequences compatible with sGBS.

### Allele-specific amplification

In a 96 well plate, 150 ng of DNA was combined with 3 pmol of M13 barcode primer (4 μL at 0.75 μM). A master mix consisting of buffer (1X final), 0.75 μL MgCl_2_ at 50 mM (2.5 mM final concentration), 1.2 μL dNTP mix at 2.5 mM for each nucleotide (200 μM final concentration for each), 0.3 pmol forward-tailed primer (0.03 μL at 10 μM: 20 nM final concentration), 3 pmol reverse primer (0.3 μL at 10 μM: 200 nM final concentration), 0.33 U Taq polymerase, and 3.62 μL H_2_O were combined with the DNA for a total volume of 15 μL for each reaction. Plates were PCR-amplified for 36 cycles consisting of 95C (1 min), 57C (20 s), and 72C (40 s). All samples in the plates were pooled and added to the quantified GBS libraries.

### Library construction and sequencing

**Figure 2:**
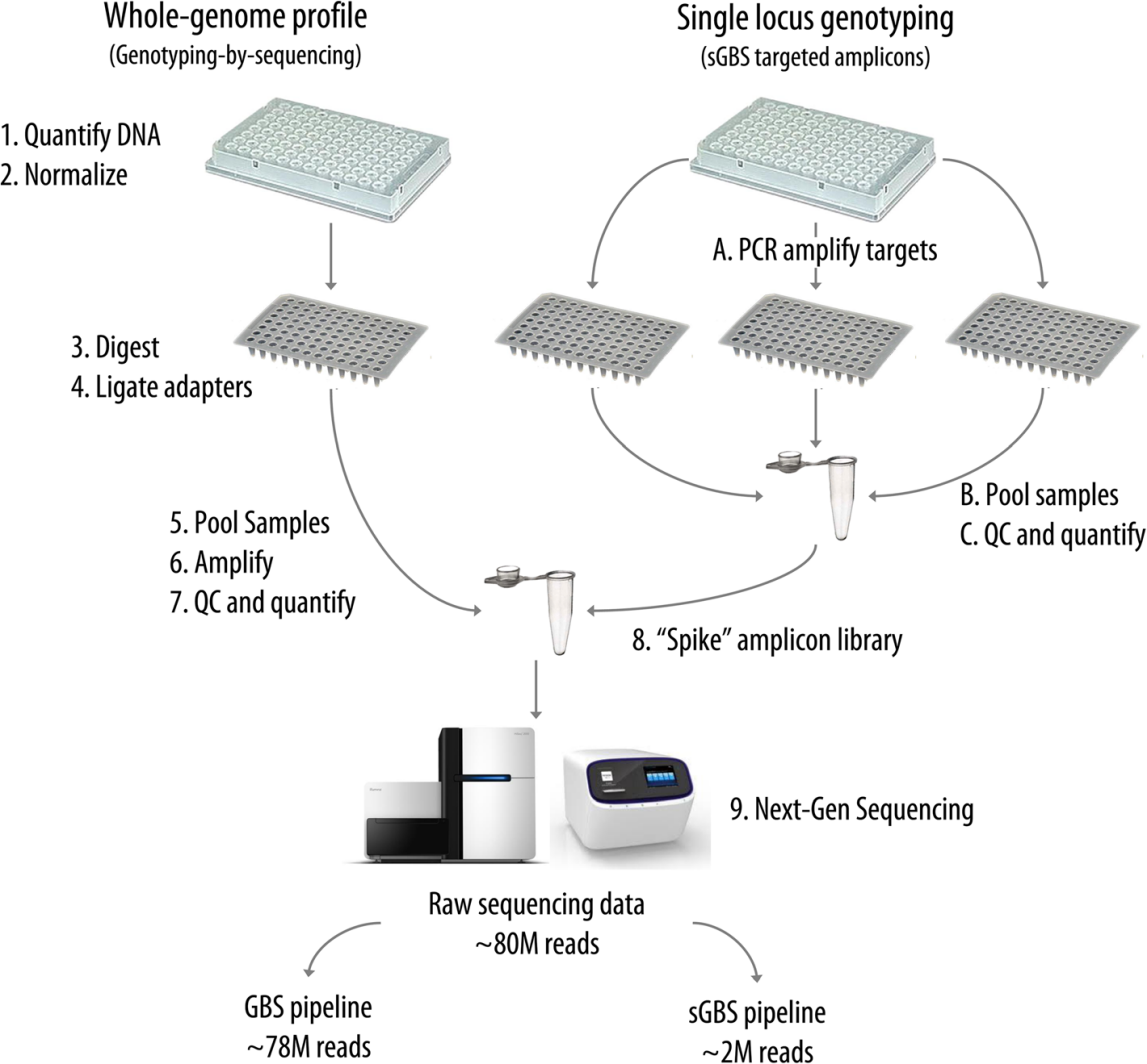
**Library construction flow chart.** GBS libraries are created following standard protocols. Each spiked library amplifies a single target locus. Spiked libraries are pooled, combined with GBS libraries, and sequenced. Sequence data for the amplicon library is parsed using the M13 and unique barcode sequence.

Two GBS libraries were prepared for Ion Torrent™ (Life Technologies, Carlsbad, CA) sequencing following the protocol from Mascher et al. [24]. Libraries were size-selected on a 2% agarose gel between 200 and 250 bp, quantified using Quant-iTTM PicoGreen® (Molecular Probes/Invitrogen Eugene, OR 97402), and normalized to 11 nM. After pooling, the amplicon libraries were quantified using PicoGreen and normalized to 1.1 nM. Five μL of the pooled amplicons were added to 50 μL of each GBS library for a final concentration of 1% (Figure 2). The libraries were prepared using the Ion PI™ Template OT2 200 Kit (v2 and v3) and then sequenced on an Ion Proton™ System using the Ion PI™ Chip Kit v1. The full protocol for library preparation is provided in Additional file 4.

### Data processing

A TASSEL pipeline designed for Illumina sequence data was modified to identify SNPs from the GBS tags [24,25]. Specifically, TASSEL was modified to process Ion Torrent sequencing sites and with variable length sequence reads. SNP genotypes were called according to the approach of Poland et al. [26] using a population-based filter. A TASSEL-based custom pipeline was written to determine the allele counts at each amplified locus by identifying the presence of both the M13 sequence and the target SNP alleles. Reads with the M13 tail sequence were parsed by barcode and the number of reads at each allele for a given locus was counted by exact matching to one of the target sequences.

### Genotype calling for allele-specific amplicons

Lines with less than 10x read coverage were not included when clustering and calling genotypes. Genotypes were called using k-means clustering and DBSCAN clustering, both performed in R [27-29]. For k-means, the relative proportion of reads for each allele were plotted to determine the appropriate number of clusters to use for this input parameter. DBSCAN relies on reachability distance to determine the appropriate number of clusters [27,28]. Varying reachability distances were empirically tested to ascertain an appropriate value. Observationally, a reachability distance of 0.1 ideally grouped all but one locus. For BS00150192, the optimal reachability distance was 0.06.

## Results and discussion

To test the approach of spiked GBS, we assayed a panel of diverse wheat lines using GBS to create a whole-genome profile and sGBS to target 11 known polymorphic sites. DNA was extracted and normalized and GBS libraries were constructed for the Ion Proton sequencing platform. The two sequenced GBS libraries contained 73 M and 81 M reads with a respective mean read length of 145 bp and 183 bp. Consistent with previous experience with unspiked GBS libraries, 83.6% and 81.3% of reads contained a good GBS barcode and a barcode plus enzyme cut site, respectively. Internal alignment-based discovery resulted in the identification of 13,617 SNPs with less than 20% missing data. This is also consistent with previous unspiked GBS libraries [24,30].

As a proportion of total sequencing output, the spiked amplicons constituted 1.8% and 3.1% of each library as determined by a count of M13 sequences. Amplicon libraries were individually analyzed to avoid bias due read number differences. For each locus, the allelic state of each line was determined by counting the number of reads containing both the sample-specific barcode and a given allele. Genotypes were called using k-means clustering in R and DBSCAN clustering using the fpc package in R [27,28]. Relative read frequency was used to group individuals into one of three classes: A, B, or Heterozygous. K-means requires a parameter specifying the number of expected clusters while DBSCAN requires the reachability distance [27]. Both of these values require individual curation for loci to ensure two (A/B or A/H) or three (A/B/H) clusters are correctly called.

**Figure 3:**
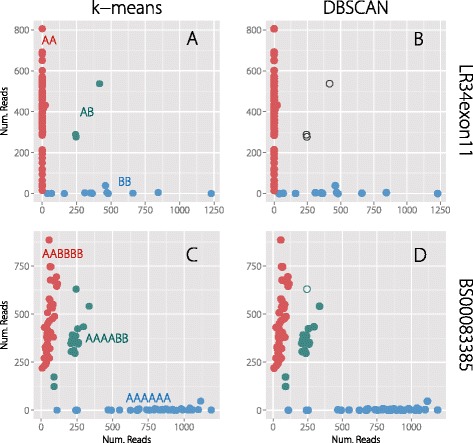
**k-means clustering and DBSCAN clustering for Lr34exon11 and BS00083385.** k-means clustering and DBSCAN clustering were used to cluster genotypes for each individual on relative read frequency of the two SNP alleles. Genotypes called within the same group are denoted by color. Unfilled symbols indicate samples that were not classified by the algorithms. **(A)** k-means and **(B)** DBSCAN clustering of LR34exon11. LR34exon11 locus is a single-copy locus and the two genotypes are easily distinguished by either clustering algorithm. Heterozygotes are characterized by an equal proportion of both alleles. **(C)** k-means and **(D)** DBSCAN clustering of BS00083385. This primer pair presumably amplifies multiple loci in the polyploid wheat genome that can still be distinguished based on relative read frequency. The three genotypic classes for individual lines are likely AAAAAA, AABBBB, and AAAABB. The BBBBBB group does not appear to be present, since a null A genotype should fall on the vertical axis with zero reads counts of allele A. DBSCAN did not classify the unfilled individual, which is potentially heterozygous at one of the loci (AAABBB).

Generally, there were few differences in the results from either method. For single-copy loci, both methods performed equally and homozygotes and heterozygotes were easily identifiable (Figure 3A). Loci with non-zero axis clusters were also easily identified with both methods. Clusters arising from multi-copy loci were often distinct enough to confidently postulate the genotype allelic state (Figure 3C). Overall, the level of concordance between the two clustering algorithms was high with 97.2% of the genotype calls the same between the two methods (Figure 3B and D). The majority of discordance was due to k-means requiring that all genotypes be classified whereas DBSCAN did not classify individuals outside of the main clusters. The DBSCAN algorithm is therefore likely of more use in polyploid species where a heterozygote may not be as readily identified (Figure 3D). Ignoring the individuals that DBSCAN did not classify, there was 100% agreement between the two methods.

Robust conversion of SNP markers between different platforms is important for future genotyping applications, but success can vary considerably [31-33]. In this study, we observed a good level of conversion from the KASP markers. Two attempted primer sets did not result in amplifying the target sequence and further efforts to optimize conditions for these primer sets were not attempted. For markers that successfully amplified, the average call rate was 94.8%. Several markers from the KASP core set resulted in non-zero axis read count clusters, likely due to the existence of homologous copies of the target locus. The percentage of alleles called for each locus and average coverage are reported in Table 1.

**Table 1:** **Reads/Marker**

**Marker**	**Call rate**	**Avg. depth**
LR34exon11	94.5%	336
Lr34intron4kasp	96.4%	114
Lr34exon12kasp	99.3%	923
LR34exon11kasp	98.7%	1573
Lr34exon22kasp	99.2%	117
BS00150192	92.8%	863
BS00089969	92.7%	564
BS00023148	98.2%	1577
BS00083385	81.0%	1118

Marker name, total call rate, and average read depth.

## Conclusions

With sGBS, we have developed a low-cost, flexible platform for whole-genome profiling and targeted, single-locus genotyping. The open architecture of primer design for the spiked amplicons enables simple inclusion of new or different target loci. Utilizing a unique set of barcodes combined with locus-specific M13 tail primers enabled sequencing of amplified targets in parallel with GBS libraries. While GBS provides a very low-cost approach for whole-genome profiling, it relies on reproducibly sequencing between restriction sites and cannot target *a priori* selected loci. Targeted amplicons fill this gap by allowing specific loci to be characterized. However, with the enormous sequencing output from current sequencing platforms, generating a sufficient number of amplicons across an appropriate number of samples to avoid unreasonable sequencing depth and cost is prohibitive. To minimize cost, we utilized a small fraction of the sequencing run (1-3%) while generating more than sufficient coverage across all target loci. Any reasonable number of amplicons could likely be combined with a GBS run. As with any sequencing approach, increasing the number of samples (or targets) decreases coverage. As sequencing output continues to increase, further ‘excess’ capacity can be leveraged in this way. However, targeted amplicon numbers beyond 10–20 are likely to be impractical relative to a fully designed array or whole-genome characterization (i.e. GBS).

Routine implementation of genotyping approaches in large genetic and breeding applications requires simple and robust laboratory pipelines. In concert with GBS library development, sGBS target amplification is a streamlined procedure affording routine, high-throughput implementation. The amplicon libraries are generated through a single PCR reaction, collectively normalized, and pooled with a GBS library. Though not attempted here, multiplex PCR reactions for the allele-specific amplification would further simplify the overall protocol.

sGBS was designed for MAS in breeding but is also broadly applicable for a large number of other molecular genetics purposes. Many approaches ranging from diversity studies [34] to genetic and association mapping [35] and genomic selection [26] have successfully applied GBS, but the number of genetic markers generated by GBS often exceeds what is needed for genetic studies, such as fine mapping or TILLING. Fine mapping for map-based cloning generally requires screening a very large population with flanking markers for the gene of interest. While GBS is not a suitable marker platform for fine mapping, utilizing the spiked portion of sGBS for these studies would be ideal. Likewise, the targeted amplicons of sGBS could also be used to screen for novel mutations in TILLING or ECO-TILLING populations. Though *a priori* SNPs were targeted in the present study, the direct sequencing of targets also enables *de novo* discovery of novel mutations as in a TILLING study.

For plant breeding, sGBS will enable breeders to genotype large collections of germplasm for specific markers by taking advantage of the massive data output of current sequencing platforms. Large numbers of markers are required for genomic selection, but plant breeders are also interested in characterizing important disease or physiological loci in breeding populations. sGBS provides a low-cost, scalable approach for both requirements and will serve as an important tool as plant breeding continues its use of molecular markers.

Since sGBS amplicons are independent of GBS libraries, breeders can generate a whole-genome profile for advanced breeding material while also applying marker-assisted selection to earlier generations. Importantly, the only realized cost for target genotyping using sGBS is a single PCR reaction. The ability to quickly identify lines containing specific alleles will enhance the capacity and speed of superior cultivar generation in breeding programs.

Plant breeding is inherently an exercise in producing and analyzing large amounts of data to discover improved rare and novel variants. Future advancements in plant breeding will fundamentally rely on new technologies being implemented that allow breeders to progress through this process with the most efficient utilization of resources and least disruption to current workflow. Plant breeding programs have historically depended on single-marker germplasm characterization and are beginning to take advantage of whole-genome profiles for genomic selection. sGBS combines both approaches, eliminating the current necessity of two distinct platforms while leveraging continual advancements in sequencing technology. This efficient strategy will allow breeders to increase the amount of germplasm and number of loci that are assayed with few changes to workflow and limited expenditure of resources. Developments like sGBS that enable genomics-assisted breeding are crucial to ensuring progress in developing improved plant varieties in the effort to eliminate hunger and poverty across the world.

## Additional files

Additional file 1: Table S1. Wheat varieties used in this analysis. Additional file 2: Table S2. Loci, target alleles, and primer sequences used for sGBS. Additional file 3: Table S3. Barcode sequences and forward oligo sequences. Additional file 4:
**Full protocol for spiked genotyping-by-sequencing.**

## Abbreviations

MAS: Marker-assisted selection
GS: Genomic selection
KASP: Kompetitive Allele specific PCR
TAS: Targeted amplicon sequencing
GBS: Genotyping-by-sequencing
