# Supplementary material for: Spiked GBS: a unified, open platform for single marker genotyping and whole-genome profiling
Source: BMC Genomics. 2015 Mar 28;16(1):248. doi: 10.1186/s12864-015-1404-9 (PMC4404221; doi:10.1186/s12864-015-1404-9)
Supplement: Additional file 4: — Full protocol for spiked genotyping-by-sequencing. [file 12864_2015_1404_MOESM4_ESM.docx]

PROTOCOL

Allele Specific Amplification using “Spiked” Genotyping-by-Sequencing

Jesse Poland

Kansas State University

Shuangye Wu

Kansas State University

Trevor Rife

Kansas State University

June 26, 2014

**Overview**

Genotyping-by-sequencing (GBS) is an approach for reduced representation sequencing of large and complex genomes. Using a restriction enzyme, a small portion of the genome can be reducibly captured and sequenced.

Often genetics research and molecular marker assisted selection in plant breeding has need for single marker assays rather than whole genome profiling.

**Primer Design**

The assay is designed as a nested PCR reaction that can be completed in a single reaction well. Each sample will have a unique barcode primer with M13(-21) tail sequence. A set of common primers for the target sequence are included that have the corresponding M13 tail on the forward primer and a tail for the reverse sequencing primer site on the reverse primer. The nested PCR reaction will produce fragments that a ready for sequencing. The sequencing read will first read through the barcode followed by the M13 sequence. The target SNP can be located directly after the forward target sequence primer or further down stream as long as it is within the read length of the sequencing platform.

**Allele Specific Amplification**

1. Normalize 5ul of DNA at 20 – 40 ng/ul in a 96 well plate
2. Add 4ul of M13 barcode primer (0.75 uM).

Note: Each sample well will have a unique barcode primer.

1. Make Master Mix for whole plate volume
2. Add 8ul of PCR master mix to samples

| Regent (Stock Concentration) | Reaction Volume (ul) | Full Plate Volume (ul) (x120) | Final Concentration |
| --- | --- | --- | --- |
| Buffer Stock (10x) | 1.5 | 180 | 1x |
| MgCl_2_ (50 mM) | 0.75 | 90 | 2.5 mM |
| dNTP mix (2.5 mM) | 1.2 | 1.2 | 200 uM (each) |
| Forward Tailed Primer (10.00 uM) | 0.03 | 3.6 | 20 nM |
| Reverse Primer (10.00 uM) | 0.3 | 36 | 200 nM |
| Taq polymerase (5.00U/ul) | 0.1 | 12 | 0.33 U |
| H_2_0 | 3.62 | 434.4 |  |
| Master Mix Total | 8 | 960 |  |
|  |  |  |  |
| DNA (20 to 40ng/ul) | 5 | - | 100 – 200 ng |
| M13 Barcode Primer (0.75 uM) | 4 | - | 200 nM |
|  |  |  |  |
| PCR reaction total volume | **15** | **-** |  |

PCR conditions

| PCR | Based pm Annealing temperature – short |  |
| --- | --- | --- |
| 1 | 95^o^C - 5 min |  |
| 2 | 95^o^C - 1 min | 36 Cycles |
| 3 | 57^o^C - 20 sec |  |
| 4 | 72^o^C - 40 sec |  |
| 6 | 72C, 10 min |  |
| 7 | 8C, forever |  |

**Spiking of Amplicon library to GBS library**

The target amplicon library should be added at a concentration of ~1% of the total GBS library.

1. Quantify GBS library using PicoGreen
2. Normalize GBS library to 50ul at 11 nM
3. Quantify amplicon library using PicoGreen
4. Normalize amplicon library to 1.1 nM
5. Add 5 ul of amplicon library to 50 ul of GBS library

| Library | Volume | Conc. | Final Conc. |
| --- | --- | --- | --- |
| GBS | 50 ul | 11 nM | 10 nM |
| Amplicon | 5 ul | 1.1 nM | 0.1 nM |
| TOTAL | 55 ul |  | 10 nM |
